# In vitro activity of oral antibiotics against extensively drug-resistant Shigella isolates: a pharmacokinetic/pharmacodynamic perspective on the intracellular pathogenesis paradigm

**DOI:** 10.1099/jmm.0.002213

**Published:** 2026-11-13

**Authors:** Dhanalakshmi Solamalai, Madhumathi Irulappan, Yamuna Devi Bakthavatchalam, Karnan Selvam, Shariqa Qureshi, Ekadashi Rajni Sabharwal, Nivedana S, Baby Abirami, Venkatalakshmipriya G, Sridhar P, Ramprakash Rayala, Kamini Walia, Balaji Veeraraghavan

**Affiliations:** 1 Department of Clinical Microbiology, Christian Medical College, Vellore, India; 2 Department of Clinical Microbiology and Infectious Diseases, Chacha Nehru Bal Chikitsalaya, Delhi, India; 3 Department of Microbiology, Mahatma Gandhi University of Medical Sciences & Technology, Jaipur, India; 4 Department of Microbiology, Rainbow Children’s Hospital, Chennai, India; 5 Descriptive Research Division, Indian Council of Medical Research, New Delhi, India

**Keywords:** epithelial invasion, extensively drug-resistant, intracellular pathogens, oral antibiotics, pharmacokinetics–pharmacodynamics, *Shigella*

## Abstract

**Introduction.** Shigellosis is emerging as a critical therapeutic challenge in low- and middle-income countries (LMICs) due to the emergence of extensively drug-resistant (XDR) strains resistant to azithromycin, third-generation cephalosporins and fluoroquinolones. The obligate intracellular lifestyle of *Shigella*, characterized by epithelial cell invasion and actin-mediated intracellular spread, limits the predictive value of conventional extracellular susceptibility testing alone.

**Hypothesis/Gap Statement.** The loss of effective oral antibiotics for XDR *Shigella* represents a major unmet clinical and public health need, particularly in endemic LMIC settings where outpatient management is essential. Current breakpoint-based susceptibility interpretations primarily reflect extracellular antimicrobial exposure and may inadequately capture the intracellular pharmacodynamic requirements of *Shigella* infection. Furthermore, integrated evaluation of *in vitro* susceptibility together with pharmacokinetic–pharmacodynamic (PK/PD) and intracellular exposure parameters for candidate oral agents against XDR *Shigella* remains limited.

**Aim.** To evaluate the molecular resistance profiles, *in vitro* activity and PK/PD applicability of candidate oral antibiotics against XDR *Shigella* isolates.

**Methodology.** Between 2010 and March 2025, 1,823 non-duplicate clinical *Shigella* isolates were screened at a tertiary care centre in South India. Fifty-two isolates fulfilled XDR criteria: azithromycin MIC ≥32 mg l^−1^, ceftriaxone MIC >4 mg l^−1^ and ciprofloxacin MIC >1 mg l^−1^. PCR assays targeted *blaCTX-M*, *blaDHA*, *blaCMY*, *blaVIM*, *blaNDM*, *mphA*, *msrE*, *ermB*, *qnrS* and *qnrB*. MICs for amoxicillin–clavulanate, cefpodoxime–clavulanate, fosfomycin, mecillinam and faropenem were determined using broth or agar dilution methods and interpreted using Clinical and Laboratory Standards Institute (CLSI) M100 (2025) or literature-derived PK/PD thresholds.

**Results.** *β*-Lactamase genes and *mphA* were detected in 96.2% of isolates, whilst *qnrS*/*qnrB* occurred in 53.8%. Cefpodoxime–clavulanate and faropenem showed 100% susceptibility, followed by fosfomycin (98.1%) and mecillinam (94.2%). MIC50/MIC90 (mg l^−1^) were 0.5/0.5 for faropenem, 0.25/2 for mecillinam and 0.25/2 for cefpodoxime–clavulanate. However, PK/PD analysis suggested that several agents lacked sustained intracellular exposure despite favourable MICs.

**Conclusion.** Favourable *in vitro* susceptibility did not consistently translate into predicted intracellular therapeutic adequacy. These findings support PK/PD-informed assessment beyond MIC-based interpretation for managing XDR shigellosis and highlight the urgent need for effective oral therapeutic options for XDR *Shigella* infections

## Introduction

Shigellosis remains a major source of morbidity and mortality in low- and middle-income countries (LMICs), accounting for an estimated 165 million episodes and over 1 million deaths annually, predominantly among children under 5 years [1, 2]. For decades, empirical oral therapy with ampicillin, cotrimoxazole, fluoroquinolones and azithromycin provided reliable outpatient management. However, the progressive emergence of multidrug-resistant (MDR) and, increasingly, extensively drug-resistant (XDR) *Shigella* strains has progressively eroded therapeutic options, rendering standard oral regimens ineffective and necessitating costly, resource-intensive intravenous carbapenem therapy unsuitable for most LMIC contexts [3–6].

Resistance patterns are geographically and temporally dynamic. In China, *Shigella sonnei* and *Shigella flexneri* with concurrent *blaCTX-M*, *mphA* and fluoroquinolone resistance determinants have driven XDR phenotypes [7, 8]. Phylogenomic analyses across Southeast Asia document the clonal expansion and regional dissemination of extended-spectrum *β*-lactamases (ESBLs) and plasmid-mediated quinolone resistance, particularly the Central Asia III fluoroquinolone-resistant *S. sonnei* lineage [8–12]. In India (2015–2019), *Shigella* resistance rates were alarming: ampicillin (68%), cotrimoxazole (75%), ciprofloxacin (61%) and ceftriaxone (45%), with multiple ESBLs (*blaCTX-M-1*, *blaTEM* and *blaOXA*), *qnrS* and *dhfr1A/sulII* confirmed among diverse plasmid backgrounds [13–17]. This regional trend mirrors global patterns, with XDR *Shigella* now documented in high-income countries, indicating trans-continental transmission and the imminent threat of untreatable dysentery worldwide [18–22].

Yet susceptibility-based algorithms traditionally applied to extracellular pathogens fundamentally misrepresent *Shigella* therapeutic challenges. Central to this framework shift is *Shigella*’s obligate intracellular lifecycle. Following low-dose colonic epithelial invasion (~10–500 bacterial cells sufficient for infection, depending on serogroup), *Shigella* spp. escape phagosomal vacuoles and exploit IcsA (VirG)-mediated actin-based motility to spread directly between epithelial cells in an actin tail-driven propulsion mechanism [23, 24]. This epithelial cell-to-cell dissemination bypasses the luminal and extracellular compartments entirely, thereby shielding the organism from both humoral immune defenses and from antibiotics whose penetration is restricted to the gut lumen or extracellular spaces. Consequently, effective antimicrobial therapy against intracellular pathogens such as *Shigella* depends not only on favourable MICs but also on sustained intracellular and epithelial drug exposure, with efficacy better predicted by pharmacokinetic–pharmacodynamic (PK/PD) indices such as time above MIC (%ƒT>MIC) and area under the concentration–time curve to MIC ratio (AUC/MIC) rather than MIC alone [25]. By correlating *in vitro* susceptibility with clinically achievable drug concentrations, PK/PD analysis improves prediction of therapeutic success, optimizes dosing strategies, supports breakpoint determination and highlights the limitations of conventional breakpoint-based susceptibility interpretations for intracellular pathogens [25, 26].

Herein, we characterize the molecular epidemiology and *in vitro* susceptibility profiles of 52 XDR *Shigella* isolates from South India against candidate oral antibiotics, then reconcile categorical susceptibility with integrated PK/PD analysis and intracellular pathophysiology to assess clinical applicability. This approach aims to complement conventional breakpoint-based interpretation by incorporating PK/PD and intracellular exposure considerations relevant to *Shigella* pathogenesis.

## Methods

### Study design and bacterial isolates

This retrospective–prospective surveillance study analysed 1,823 non-duplicate *Shigella* spp. clinical isolates recovered from stool and blood cultures between January 2010 and March 2025 at a tertiary care hospital in Vellore, South India. Archived isolates (2010–2022; *n*=1,573) were retrieved from the Department of Microbiology repository (stored at −80 °C in glycerol stocks), whilst prospective isolates (2023–March 2025; *n*=250) were processed fresh. As routine azithromycin susceptibility testing was not performed before 2017, retrospective identification of XDR isolates from the pre-2017 period was based on available archived antimicrobial susceptibility testing (AST) records for ceftriaxone and ciprofloxacin resistance, together with whole-genome sequencing (WGS)-based detection of azithromycin resistance determinants associated with the XDR phenotype. In addition, *Shigella* isolates suspected to be XDR were also received from centres across India, of which six were confirmed as XDR and were included in the study. Isolates were identified to species and serogroup level using standard biochemical tests (triple sugar iron and Indole test, Methyl Red test, Voges-Proskauer test,Citrate utilization test [IMViC]), slide agglutination with commercial antisera (Denka Seiken, Tokyo, Japan), and confirmed by matrix-assisted laser desorption/ionization-time of flight (MALDI-TOF) MS (Bruker Daltonics) where required.

XDR status was defined operationally as concurrent resistance to the following: (i) azithromycin (MIC ≥32 mg l^−1^), (ii) third-generation cephalosporins (ceftriaxone; MIC >4 mg l^−1^) and (iii) fluoroquinolones (ciprofloxacin; MIC >1 mg l^−1^). XDR screening was performed via archived AST records, followed by prospective confirmation on all 52 XDR isolates. The study adheres to STROBE guidelines for observational research.

### Antimicrobial susceptibility testing

XDR phenotypes were confirmed and MICs determined for candidate oral agents using standard methodologies aligned with CLSI M100 35th edition (2025) guidelines for Enterobacterales [26]. Disk diffusion (Kirby–Bauer) preceded quantitative MIC testing for screening purposes. Broth microdilution (BMD) using Sensititre^®^ TREK custom-configured panels (Trek Diagnostic Systems, Thermo Fisher Scientific, Waltham, MA, USA) was employed for azithromycin, ceftriaxone, cefpodoxime, ciprofloxacin and meropenem (confirmation of resistance profile). For candidate oral agents, MICs were determined as follows: amoxicillin–clavulanate (2 : 1 ratio) and cefpodoxime–clavulanate (fixed 4 µg ml^−1^ clavulanate): BMD in cation-adjusted Mueller–Hinton broth II (Thermo Fisher Scientific) using 96-well panels, MIC ranges 0.016–256 mg l^−1^; fosfomycin (trometamol): BMD supplemented with glucose-6-phosphate (50 µg ml^−1^, essential cofactor for fosfomycin uptake), MIC ranges 0.016–256 mg l^−1^; faropenem: BMD as above, MIC ranges 0.016–256 mg l^−1^; mecillinam: agar dilution using Mueller–Hinton agar (Thermo Fisher Scientific) with 2% NaCl, MIC ranges 0.016–256 mg l^−1^ (agar dilution preferred for mecillinam per CLSI recommendations).

All testing plates were incubated aerobically at 35±2 °C for 16–20 h. Quality control strains included *Escherichia coli* ATCC 25922 and *Klebsiella pneumoniae* ATCC 700603, tested in parallel per CLSI guidelines. As *Shigella*-specific interpretive breakpoints are unavailable for most of the evaluated antibiotics, susceptibility results were interpreted using CLSI M100 (2025) Enterobacterales breakpoints, consistent with current World Health Organization (WHO)- and European Committee on Antimicrobial Susceptibility Testing (EUCAST)-aligned *Shigella* surveillance practices. Fosfomycin MICs were interpreted using CLSI Enterobacterales criteria with glucose-6-phosphate supplementation incorporated during testing. Faropenem lacks CLSI-validated breakpoints for *Shigella*; therefore, interpretation was based on published PK/PD-derived susceptibility thresholds (MIC ≤2 mg l^−1^) from Monte Carlo simulation and clinical pharmacodynamic studies [27, 28].

As conventional susceptibility categorization based on CLSI/EUCAST breakpoints is primarily derived for extracellular bacterial infections, interpretation of activity against *Shigella* requires additional consideration of PK/PD parameters relevant to intracellular exposure. Therefore, published PK/PD data, including tissue penetration, intracellular accumulation, probability of target attainment (PTA) and established exposure indices (%ƒT>MIC and AUC/MIC), were integrated with the MIC distributions generated in this study to assess the potential clinical applicability of candidate oral agents.

### Molecular detection of resistance genes

Genomic DNA was extracted via boiling lysis: isolates were suspended in sterile distilled water, heated at 100 °C for 10 min and centrifuged (10,000 ***g***, 5 min), and the resulting supernatant was used directly as DNA template. PCR targeting resistance genes was performed as multiplex or singleplex assays using primer pairs (Table S1, available in the online Supplementary Material) designed to amplify the following: ESBL/AmpC genes: *blaCTX-M-1* group (CTX-M-1, -3, -10, -14, -15, -27, -32 and -55), *blaDHA*, *blaCMY* and *blaMOX*; carbapenemase genes: *blaVIM* and *blaNDM*; macrolide resistance genes: *mphA*, *mphE*, *ermB* and *msrE*; plasmid-mediated quinolone resistance genes: *qnrS* (alleles S1/S2/SC) and *qnrB*. PCR cycling conditions used in this study are: initial denaturation at 95 °C for 5 min; 35 cycles of 95 °C for 30 s, 55–60 °C (primer-specific) for 30 s and 72 °C for 30 s; final extension at 72 °C for 10 min. Amplicons were visualized on 1.5–2% agarose gels stained with ethidium bromide under UV transillumination. Known gene-carrying in-house reference strains served as positive controls; sterile water served as a negative control.

## Results

### Isolate population and XDR prevalence

Among 1,823 non-duplicate *Shigella* spp. isolates collected between January 2010 and March 2025, including strains received from collaborating hospitals, 52 isolates (5%; 52 out of 1,084 isolates received from 2015 to March 2025) met the criteria for extensive drug resistance (XDR), defined by concurrent resistance to azithromycin (MIC ≥32 mg l^−1^), third-generation cephalosporins (ceftriaxone MIC >4 mg l^−1^) and fluoroquinolones (ciprofloxacin MIC >1 mg l^−1^). The earliest XDR isolates, identified retrospectively from 2015, were confirmed through review of archived AST data, WGS-derived resistance profiles [17] and prospective AST reconfirmation. The XDR isolate collection comprised primarily *S. flexneri* (*n*=31; 59.6%) and *S. sonnei* (*n*=21; 40.4%), consistent with regional epidemiological trends.

### Molecular characterization of resistance determinants

PCR analysis of the 52 XDR *Shigella* isolates revealed high prevalence of key resistance genes with excellent phenotype–genotype concordance. ESBL and AmpC determinants (*blaCTX-M*, *blaDHA-1* and *blaCMY-1*) were detected in 50 out of 52 (96.2%) isolates, accounting for third-generation cephalosporin resistance. *blaCTX-M* was the predominant ESBL type (identified in 15 of 27 ESBL-positive isolates where genotyping to subgroup was performed), consistent with global patterns. These genes were frequently embedded on diverse plasmid backbones, facilitating horizontal gene transfer.

Carbapenemase genes (*blaVIM* and *blaNDM*) were identified in 2 out of 52 (3.8%) meropenem-non-susceptible isolates, confirming the molecular basis for discordant phenotypic carbapenem resistance in the minority. Macrolide resistance, predominantly mediated by *mphA* (50/52; 96.2%), occasionally co-occurring with *ermB* (2/52; 3.8%), explained azithromycin resistance phenotypes. Sporadic detection of *msrE* (1/52) and *mphE* (1/52) highlighted the diversity of macrolide resistance mechanisms. Plasmid-mediated quinolone resistance genes *qnrS* and *qnrB* were present in 28 out of 52 (53.8%) isolates. Chromosomal QRDR (quinolone resistance-determining region) mutations (*gyrA* and *parC*) were not assessed in this study, limiting complete characterization of fluoroquinolone resistance architecture.

Genotype–phenotype analysis showed complete concordance between acquired resistance determinants and corresponding *β*-lactam and macrolide resistance phenotypes, including *blaCTX-M*/*blaDHA*/*blaCMY* with ceftriaxone resistance and *mphA/ermB/msrE/mphE* with azithromycin resistance. Quinolone resistance genes (*qnrS/qnrB*) were detected in 53.8% of isolates, whilst resistance in the remaining isolates was likely chromosomal (*gyrA/parC* not evaluated). A summary of the PCR-based molecular typing of antibiotic resistance genes detected in the clinical isolates is provided in Table S2.

### Antimicrobial susceptibility testing

MICs for the five candidate oral agents (amoxicillin–clavulanate, mecillinam, cefpodoxime–clavulanate, fosfomycin and faropenem) were determined for all 52 XDR isolates. Results are presented in Table 1(a) (MIC distribution) and Table 1(b) (categorical interpretation using CLSI 2025 Enterobacterales breakpoints, except faropenem per literature thresholds) [27, 28].

**Table 1.** (A) MIC distribution, MIC₅₀/MIC₉₀ values and categorical susceptibility profiles of candidate oral antibiotics (mecillinam, amoxicillin–clavulanate, cefpodoxime–clavulanate, faropenem and fosfomycin) against XDR *Shigella* clinical isolates, interpreted using CLSI 2025 breakpoints. Colour coding denotes susceptibility status (green, susceptible; yellow, intermediate; red, resistant). (B) Summary of susceptibility breakpoints as per CLSI 2025 guideline against amoxicillin–clavulanate, mecillinam, cefpodoxime–clavulanate, fosfomycin and faropenem

A					
Antibiotic concentration (µg ml^−1^)	Amoxicillin/clavulanate	Mecillinam	Cefpodoxime/clavulanate	Fosfomycin	Faropenem
0.008					
0.016					
0.031		3			
0.063		1			
0.125		12	21		1
0.250		15	6	1	16
0.500		7	5	1	31
1	9	2	13	7	4
2	8	8	7	11	
4	23	2		6	
8	2			7	
16	6	1		7	
32	3			11	
64	2	1			
128				1	
MIC_50_	4 µg ml^−1^	0.25 µg ml^−1^	0.25 µg ml^−1^	4 µg ml^−1^	0.5 µg ml^−1^
MIC_90_	16 µg ml^−1^	2 µg ml^−1^	2 µg ml^−1^	32 µg ml^−1^	0.5 µg ml^−1^
% susceptible	78.84	96.15	100	98.07	100
% intermediate	11.53	1.92		1.92	
% resistant	9.61	1.92			
**B**					
**Antibiotic**	**Susceptible**	**Intermediate**	**Resistant**		
Amoxicillin/clavulanate	≤8	16	≥32		
Mecillinam	≤8	16	≥32		
Cefpodoxime/clavulanate	≤2	4	≥8		
Fosfomycin	≤64	128	≥256		
Faropenem	No interpretation				

Susceptibility testing revealed distinct activity profiles among the evaluated oral antimicrobial agents. Amoxicillin–clavulanate demonstrated variable activity, with 41 out of 52 isolates (78.8%) categorized as susceptible, 6 out of 52 (11.5%) as intermediate and 5 out of 52 (9.6%) as resistant. The MIC distribution was right-shifted, with an MIC₅₀ of 4 µg ml^−1^ and an MIC₉₀ of 16 µg ml^−1^, indicating reduced inhibitory margins and limiting its reliability for empirical therapy. In contrast, mecillinam exhibited high *in vitro* activity, with 49 out of 52 isolates (94.2%) susceptible, 1 out of 52 (1.9%) intermediate and only 2 out of 52 (3.8%) resistant. This was supported by low MIC values (MIC₅₀=0.25 µg ml^−1^; MIC₉₀=2 µg ml^−1^), reflecting superior potency compared with amoxicillin–clavulanate. Cefpodoxime–clavulanate showed uniform efficacy, with all isolates (52/52, 100%) categorized as susceptible. The MIC distribution was narrow, with an MIC₅₀ of 0.25 µg ml^−1^ and an MIC₉₀ of 2 µg ml^−1^, indicating consistent restoration of cephalosporin activity by *β*-lactamase inhibition.

Fosfomycin retained high categorical susceptibility (51/52, 98.1%), with only one isolate (1.9%) classified as intermediate. However, the MIC distribution was comparatively right-shifted (MIC₅₀=4 µg ml^−1^; MIC₉₀=32 µg ml^−1^), representing an eightfold increase above standard urinary tract infection breakpoints and suggesting reduced inhibitory margins despite preserved categorical susceptibility.

Faropenem demonstrated the most consistent activity, with all isolates (52/52, 100%) susceptible using a literature-defined breakpoint of ≤2 mg l^−1^. Both MIC₅₀ and MIC₉₀ were 0.5 µg ml^−1^, indicating an exceptionally tight MIC distribution and uniform *in vitro* potency across the isolate collection.

Collectively, cefpodoxime–clavulanate, faropenem and mecillinam emerged as the most potent agents *in vitro*, with ≥94% susceptibility and MIC₉₀ values ≤2 µg ml^−1^. These agents exhibited superior activity and tighter MIC distributions relative to amoxicillin–clavulanate and fosfomycin, which showed broader MIC ranges and elevated MIC₉₀ values. However, categorical susceptibility alone provides insufficient clinical guidance.

## Discussion

### The intracellular pathogenesis paradigm: reframing susceptibility

*Shigella* bacillary dysentery is frequently conceptualized as a conventional enteric infection treatable through standard antimicrobial principles. This framing fundamentally obscures *Shigella*’s defining pathogenic feature: obligate intracellular epithelial cell-to-cell dissemination that bypasses luminal and extracellular infection phases entirely. Following invasion of colonic epithelial cells through an M-cell-dependent transcytosis pathway or direct epithelial disruption, *Shigella* spp. escape the initial phagosomal vacuole through secretion of IcsA (invasion plasmid antigen C, also designated VirG), a bacterial outer membrane protein that recruits host actin filaments [23, 29]. This actin-based propulsion system drives bacterial migration directly through the cytoplasm and into adjacent epithelial cells, enabling rapid cell-to-cell spread and establishing a persistent intracellular infection focus that evades both luminal antibiotics and humoral immune defences [30, 31].

This intracellular lifestyle has profound therapeutic implications. Clinical efficacy depends not simply on *in vitro* susceptibility, a metric derived from extracellular bacterial growth, but on the capacity of antimicrobial agents to achieve sustained intracellular concentrations within epithelial cells and tissue macrophages sufficient to maintain bactericidal or bacteriostatic activity. Accordingly, PK/PD indices such as free time above MIC (%ƒT>MIC) and AUC/MIC must be evaluated in the context of intracellular penetration and compartmental distribution, not plasma or luminal MICs alone [25]. To contextualize these parameters across the evaluated oral agents, an integrated PK/PD and intracellular relevance framework is summarized in Table 2.

**Table 2.** Integrated PK/PD and intracellular-relevance assessment of oral agents for XDR *Shigella*.

Drug	Adult oral dose	MIC₉₀ (mg l^−1^)*	Oral bioavailability	Tissue/epithelial penetration	Intracellular/phagocyte activity	Dominant PK/PD driver	Published PK/PD/PTA evidence	Available clinical evidence	Integrated PK/PD–pathogenesis assessment	Inferred clinical applicability	Ref
Pivmecillinam (mecillinam)	400–800 mg TID	2	High (~70%)	Moderate tissue penetration with high luminal and urinary exposure	Limited intracellular accumulation; no documented intracellular bactericidal activity	%ƒT>MIC (≥40–50%)	Adequate PTA for low-MIC extracellular isolates; short half-life may restrict sustained epithelial exposure	Limited evidence in invasive shigellosis; primarily evaluated in urinary and non-invasive enteric infections	Favourable luminal exposure but limited intracellular durability following epithelial invasion	Potential utility in mild/non-invasive disease; limited role in invasive XDR shigellosis	[46–51]
Amoxicillin–clavulanate	500/125 mg q8 h or 875/125 mg q12 h	16	Moderate (~60%)	Moderate tissue distribution with reduced exposure at elevated MICs	Minimal intracellular accumulation and absent documented intracellular activity	%ƒT>MIC (≥30–40%)	Monte Carlo simulations demonstrate acceptable PTA only for low-MIC isolates (≤0.5 mg l^−1^)	Historical clinical use with limited evidence in contemporary XDR *Shigella* infection	Elevated MIC₉₀ and limited intracellular exposure reduce likelihood of reliable PK/PD target attainment	Limited suitability for invasive XDR infection	[52–58]
Cefpodoxime–clavulanate	200 mg q12 h	2	Moderate (~50%)	Moderate intestinal and epithelial tissue penetration	Limited phagocyte penetration and negligible intracellular accumulation	%ƒT>MIC (~40%)	PTA acceptable for susceptible low-MIC isolates; clavulanate dosing discordance may compromise sustained exposure	Sparse direct evidence in XDR *Shigella* infection	Favourable MIC profile offset by limited intracellular exposure and PK mismatch	Potential option for mild disease with confirmed susceptibility	[59–65]
Faropenem	200–300 mg q8 h	0.5	High (70–80%)	Good epithelial and systemic tissue distribution	Functional intracellular exposure reported despite limited true accumulation	%ƒT>MIC (>40%)	Published PK/PD and Monte Carlo simulation studies support favourable PTA at low MICs	Emerging PK/PD and clinical evidence; no controlled shigellosis trials available	Most favourable balance of MIC profile, tissue penetration and exposure durability among evaluated oral agents	Most promising oral candidate; requires prospective clinical validation	[66–74]
Fosfomycin (trometamol)	3 g q24–48 h	32	Variable (34–58%)	Excellent tissue and interstitial penetration	Documented intracellular activity with exposure-dependent killing	AUC/MIC	≥90% PTA declines substantially at MICs>4 mg l^−1^; resistance emergence reported during monotherapy	Limited evidence in invasive enteric infection	Favourable intracellular penetration offset by elevated MIC₉₀, variable absorption and resistance risk	Unsuitable as reliable monotherapy; possible adjunctive role	[75–86

* This study.

This paradigm explains the consistent clinical success of fluoroquinolones and azithromycin in XDR shigellosis. Fluoroquinolones achieve tissue: plasma concentration ratios of 5–20 and intracellular accumulation 10–50-fold above plasma levels, with prolonged post-antibiotic effects enabling bacterial suppression even after drug washout [32, 33]. Azithromycin exhibits exceptional intracellular penetration, with macrophage uptake ratios (intracellular: extracellular) exceeding 100-fold and sustained intracellular persistence [34–36]. Conversely, agents demonstrating favourable *in vitro* MICs but lacking intracellular pharmacokinetics, such as amoxicillin–clavulanate, cefpodoxime–clavulanate and even fosfomycin, frequently fail clinically despite categorical susceptibility (Table 2).

### Molecular epidemiology and resistance architecture

The detection of *blaCTX-M*/*blaDHA*/*blaCMY* in 96.2% of XDR isolates, predominantly *blaCTX-M-1* group ESBLs, aligns with our earlier observation [15] North Indian surveillance (61% ESBL prevalence) [13] and Bangladesh phylogenomics documenting *blaCTX-M-15*-harbouring *S. sonnei* clones [9]. Macrolide resistance via *mphA* (96.2%) reflects global aquatic and clinical reservoir dissemination of this horizontally transferable determinant. Fluoroquinolone resistance genes (*qnrS/qnrB*; 53.8%) co-occurring with chromosomal QRDR mutations (not characterized here) explain the phenotypic fluoroquinolone resistance observed. These molecular patterns establish a robust framework for real-time PCR-based surveillance, plasmid tracking and outbreak containment, essential for timely stewardship interventions in resource-limited settings.

### PK/PD deconstruction of oral agents

Despite promising MICs, integration of pharmacokinetic data and intracellular pathophysiology reveals critical exposure gaps as follows: mecillinam (pivmecillinam) selectively inhibits penicillin-binding protein 2 (PBP2), unique among *β*-lactams for targeting Gram-negative morphology and rendering it bactericidal against organisms resistant to cell wall synthesis inhibition via PBP1/3. Resistance is mainly chromosomal and linked to stress-response and cell wall pathway mutations rather than classical *β*-lactamase production (Table 3). *In vitro* efficacy against XDR *Shigella* (MIC_90_=2 µg ml^−1^) mirrors reports of superior activity against *S. flexneri* (MIC_90_=1 µg ml^−1^) over *S. sonnei* (MIC_90_=4 µg ml^−1^) [37]. However, oral pivmecillinam achieves peak plasma concentrations of 1.7–2.5 mg l^−1^, with a plasma half-life of ~1 h, creating a brief, transient exposure window [38, 39]. Moreover, whilst luminal and urinary concentrations are extraordinarily high (owing to high renal clearance and biliary/faecal excretion), intracellular penetration into epithelial cells and macrophages is negligible, and documented intracellular activity is absent (Table 2). For invasive shigellosis requiring sustained epithelial cell penetration, this compartmental imbalance renders mecillinam therapeutically inadequate despite favourable luminal activity suitable for non-invasive lower urinary tract infection.

**Table 3.** Antibiotics, molecular targets, resistance mechanisms and references

Antibiotic	Molecular target	Resistance mechanism	References
**Pivmecillinam (mecillinam)**	PBP2	Mutations in *mrdA*, *spoT*, aminoacyl-tRNA synthetases; clinically important resistance mainly via cysB-mediated stress adaptation (phenotypically suppressed in urine)	[87, 88]
**Cefpodoxime/clavulanate**	PBP1 and PBP3	TEM-1 *β*-lactamase; porin alterations; OXA-30 *β*-lactamase; chromosomal AmpC overexpression/promoter mutations; ESBL+AmpC synergy	[89–91]
**Amoxicillin/clavulanate**	PBP1A, PBP4 (also PBP2 and PBP3 in *H. pylori*)	OXA-1 *β*-lactamase; hyperproduction of penicillinase; plasmidic and chromosomal AmpC; inhibitor-resistant TEM (IRT); promoter-driven *β*-lactamase overexpression	[91–96]
**Fosfomycin**	MurA (UDP-*N*-acetylglucosamine enolpyruvyl transferase)	Chromosomal mutations in *murA*, *glpT*, *uhpT*; plasmid-mediated enzymes fosA, fosE, fosF, fosG, fosH	[97–100]
**Faropenem**	Highest affinity for PBP2, followed by PBP1A, PBP1B, PBP3, PBP4	ESBL production (especially CTX-M) with porin loss; hydrolysis by serine and metallo-*β*-lactamases; reduced outer-membrane permeability; cross-resistance with carbapenems	[101–105]

IRT, inhibitor-resistant TEM.

Faropenem medoxomil (oral ester prodrug) provides substantially improved bioavailability (70–80%) compared to faropenem sodium (20–30%), achieving peak plasma concentrations of 13–14 mg l^−1^ [40]. Faropenem shows highest affinity for PBP2 with additional binding to PBP1A/1B and PBP3, resulting in potent inhibition of cell wall synthesis. Resistance is mediated by *β*-lactamase activity combined with reduced membrane permeability and may overlap with carbapenem resistance mechanisms (Table 3). PK/PD modelling demonstrates >90% PTA for a bacteriostatic %ƒT>MIC target (~20%) only for isolates with MICs≤0.06 mg l^−1^ [41]. In the present study, MIC_90_=0.5 mg ml^−1^ represents an 8.3-fold excess above the optimal PTA threshold, exceeding reliable %ƒT>MIC target attainment. Furthermore, whilst faropenem achieves reasonable tissue distribution, documented intracellular accumulation in epithelial cells or macrophages is minimal (Table 2). The apparent low MIC_90_ masks a fundamental PK/PD mismatch for epithelial cell infections.

Amoxicillin–clavulanate, despite exhibiting reasonable tissue distribution (serum:tissue penetration ratio ~1 : 1), amoxicillin–clavulanate demonstrates negligible intracellular accumulation and absent documented intracellular bactericidal activity. Amoxicillin binds multiple PBPs (PBP1A, PBP2, PBP3 and PBP4) to inhibit peptidoglycan synthesis, with clavulanate extending activity against *β*-lactamase producers. Resistance is largely due to *β*-lactamase hyperproduction and inhibitor-resistant enzymes, producing quantitative MIC shifts (Table 3). Monte Carlo simulations indicate ≥90% PTA for a bactericidal %ƒT>MIC target (40%) only for isolates with MICs≤0.5 mg l^−1^ with a 500/125 mg three-times-daily regimen, reducing to 0.25 mg l^−1^ with twice-daily 875/125 mg dosing (Table 2). With an observed MIC_90_=16 µg ml^−1^, 32–64-fold above optimal thresholds, reliable pharmacodynamic target attainment is unattainable at standard doses [42]. This unfavourable PK/PD profile, compounded by absent intracellular activity, renders amoxicillin–clavulanate a poor option for invasive XDR shigellosis.

Cefpodoxime inhibits cell wall synthesis through binding to PBP1 and PBP3, whilst clavulanate restores activity by inhibiting class A *β*-lactamases. Resistance involves *β*-lactamase production, porin alterations and AmpC overexpression (Table 3). Cefpodoxime achieves moderate intestinal tissue penetration, yet phagocyte entry and intracellular accumulation remain limited. A critical pharmacologic mismatch exists: cefpodoxime is typically dosed twice daily (200 mg q12 h), whilst clavulanate is optimally dosed three times daily. This discordance compromises sustained %ƒT>MIC target attainment against higher-MIC isolates (Table 2). Although categorical susceptibility is uniform (MIC_90_=2 µg ml^−1^), the absence of intracellular activity and the dosing mismatch restrict utility to mild, low-MIC disease with confirmed susceptibility, rendering it unsuitable for empirical therapy of invasive infection [43, 44].

Fosfomycin inhibits MurA, blocking the first step of peptidoglycan biosynthesis. Resistance arises through mutations in *murA* or uptake systems (*glpT* and *uhpT*) and by plasmid-mediated fos genes that inactivate the drug (Table 3). Fosfomycin demonstrates excellent tissue penetration and documented intracellular activity via exposure-dependent (AUC/MIC-driven) bactericidal mechanisms. Oral fosfomycin trometamol achieves peak plasma concentrations of 22–32 mg l^−1^, with ≥90% PTA for AUC/MIC=23 target only for isolates with MICs ≤4 mg ml^−1^ [45]. In this study, MIC_90_=32 µg ml^−1^, eightfold above the optimal threshold, precludes reliable PK/PD target attainment (Table 2). Furthermore, fosfomycin exhibits rapid resistance emergence under monotherapy pressure and is hydrophilic with variable intestinal absorption [42, 43]. Whilst intracellular activity is theoretically advantageous, high MICs, resistance risk and variable bioavailability preclude reliable monotherapy for invasive XDR shigellosis.

Among oral agents, tebipenem pivoxil (an orally absorbed carbapenem ester prodrug) provides the most favourable systemic pharmacokinetics, achieving higher and more sustained plasma concentrations than other oral *β*-lactams. Documented *in vitro* activity against MDR/XDR *Shigella* spp. and superior %ƒT>MIC target attainment supports its evaluation as a potential step-down alternative to intravenous carbapenems. A randomized phase IIb trial (NCT05121974) is actively recruiting to evaluate oral tebipenem pivoxil against intravenous ceftriaxone for shigellosis in children. However, the prospect of widespread oral carbapenem deployment raises justified concerns regarding amplification of carbapenem-resistant organisms and accelerated resistance emergence, necessitating strict stewardship protocols, restricted use to controlled clinical settings and surveillance integration.

### Clinical translation: reconciling *in vitro* promise with pathophysiological reality

The present data expose a critical disconnect between categorical susceptibility and clinical applicability. Cefpodoxime–clavulanate, faropenem and mecillinam achieve 94–100% categorical susceptibility and MIC_90_ ≤2 µg ml^−1^, metrics that, applied to extracellular pathogens, would herald therapeutic optimism. Yet integration of intracellular pathophysiology reveals that these agents, with the possible exception of faropenem at its lowest MICs, lack the sustained intracellular exposure requisite for eradicating *Shigella* spp. after epithelial invasion. Luminal-biassed agents (mecillinam, cefpodoxime–clavulanate and amoxicillin–clavulanate) provide excellent extracellular suppression but fail against intracellular foci. Fosfomycin, despite intracellular activity potential, is undermined by high MICs and resistance risk.

This explains why Centers for Disease Control and Prevention (CDC) guidance restricts pivmecillinam and other oral agents to step-down therapy after intravenous carbapenem induction, and why guidelines universally recommend hospitalization for XDR shigellosis. Precision-guided strategies should incorporate rapid molecular diagnostics for real-time pathogen identification and antimicrobial resistance prediction; PK/PD-optimized dosing to maximize intracellular drug exposure; combination therapy to achieve synergistic intracellular activity; and integrated clinical–microbiological endpoints.

## Limitations

The PK/PD assessment was derived from published pharmacokinetic data, Monte Carlo simulation studies and observed MIC distributions rather than original population PK modelling or clinical outcome analysis. Intracellular activity and PTA were inferred from published literature without experimental validation in *Shigella*-specific infection models. Additionally, *gyrA* and *parC* mutations were not systematically analysed, limiting complete characterization of quinolone resistance, whilst the predominantly single-centre isolate collection may not fully represent broader XDR *Shigella* epidemiology.

## Conclusion

Analysis of 52 XDR *Shigella* isolates from South India reveals a fundamental disconnect between *in vitro* susceptibility and expected clinical performance. Despite favourable MIC₉₀ values (≤2 µg ml^−1^) and high CLSI susceptibility rates, efficacy depends on achieving adequate exposure within epithelial and phagocytic compartments. Mecillinam lacks the intracellular pharmacokinetic durability needed to limit cell-to-cell spread, whilst cefpodoxime–clavulanate, although potentially adequate for low-MIC isolates, is constrained by poor phagocyte penetration. Faropenem offers the most credible oral PK/PD profile given its tissue distribution, phagocyte entry and low MIC₉₀, yet *in vivo* intestinal and clinical data in shigellosis remain limited. Amoxicillin–clavulanate and fosfomycin are further restricted by elevated MIC demands or exposure variability. Tebipenem pivoxil is promising but risks accelerating carbapenem resistance, mandating urgent clinical evaluation under strict stewardship. Collectively, these findings call for a shift from MIC-based categorization to PK/PD-guided therapy that explicitly incorporates intracellular exposure, supported by strengthened surveillance, exposure optimization and vaccine acceleration. Collectively, this study supports the need to complement conventional MIC-based interpretation with PK/PD-informed assessment approaches that consider intracellular pathogen biology, whilst emphasizing that prospective clinical studies are essential before translating these observations into therapeutic recommendations.

## Supplementary material

10.1099/jmm.0.002213 Supplementary Material 1.

## Abbreviations

AST: antimicrobial susceptibility testing
AUC/MIC: area under the concentration–time curve to MIC ratio
BMD: broth microdilution
CDC: Centers for Disease Control and Prevention
CLSI: Clinical and Laboratory Standards Institute
ESBLs: extended-spectrum *β*-lactamases
EUCAST: European Committee on Antimicrobial Susceptibility Testing
LMICs: low- and middle-income countries
MALDI-TOF: matrix-assisted laser desorption/ionization-time of flight
MDR: multidrug-resistant
PBP2: penicillin-binding protein 2
PK/PD: pharmacokinetic/pharmacodynamic
PTA: probability of target attainment
QRDR: quinolone resistance-determining region
WGS: whole-genome sequencing
WHO: World Health Organization
XDR: extensively drug-resistant
