# Supplementary material for: In vitro activity of oral antibiotics against extensively drug-resistant Shigella isolates: a pharmacokinetic/pharmacodynamic perspective on the intracellular pathogenesis paradigm
Source: J Med Microbiol. 2026 Nov 13;75(11):002213. doi: 10.1099/jmm.0.002213 (PMC13765529; doi:10.1099/jmm.0.002213)
Supplement: Supplementary file 1 [file jmm-75-02213-s001.pdf]

Supplementary Table 1: List of Primers used in this study to type the molecular determinants of the antibiotic resistance observed among the clinical isolates analyses in this study

Gene	Primer	Primer Sequence (5'- >3')	Amplicon Size	Tm	Reference/ Source
mphE	mphE_F	GCAATTGGAAACCCGCTACA	177	58	In-house
	mphE_R	GCAAAATCAATCGCTGGGTC			
ermB	ermB_F	GAAAAAGTACTCAACCAAATA	425	45	In-house
	ermB_R	AAT TTA AGTACCGTTACT			
msrE	msrE_F	TATAGCGACTTTAGCGCCAA	395	56	In-house
	msrE_R	GCCGTAGAATATGAGCTGAT			
mphA	mphA_F	GTGAGGAGGAGCTTCGCGAG	403	60	In-house
	mphA_R	TGCCGCAGGACTCGGAGGTC			
CTXM-group 2	CTX-M-group 2_F	CGTTAACGGCACGATGAC	404	60	In-house
	CTXM-group 2_R	CGATATCGTTGGTGGTRCCAT			
CTX-M14	CTX-M14_F	TCAAGCCTGCCGATCTGGT	561	60	In-house
	CTX-M14_R	TGATTCTCGCCGCTGAAG			
CTX-M15	CTX-M15_F	TTAGGAARTGTGCCGCTGYA	688	60	In-house
	CTX-M15_R	CGATATCGTTGGTGGTRCCCAT			
DHA-1	DHA-1_F	AACTTTCACAGGTGTGCTGGGT	405	60	In-house
	DHA-1_R	CCGTACGCATACTGGCTTTGC			
CMY-4	CMY_F	CGAAGAGGCAATGACCAGAC	538	60	In-house
	CMY_R	ACGGACAGGGTTAGGTTAGGATAGY			
VIM	VIM_F	GATGGTGTTTGGTCGCATA	390	54	Dallenne et al., 2011
	VIM_R	CGAATGCGCAGCACCAG			
NDM	NDM_F	GGTTTGGCGATCTGGTTTTC	621	54	Poirel et al., 2011
	NDM_R	CGGAATGGCTCATCACGATC			
qnrS	qnrS-F	ACGACATTCGTCAACTGCAA	417	53	In-house
	qnrS-R	TAAATTGGCACCCTGTAGGC			
qnrB	qnrB_F	GATCGTGAAAGCCAGAAAGG	469	53	In-house
	qnrB_R	ACGATGCCTGGTAGTTGTCC			

		**Supplementary Table 2:** Summary of the PCR analyses aimed to type the molecular determinants of antibiotics resistance observed in the clinical isolates											
**Isolate No**		**Macrolide**				**Ciprofloxacin**		**Third-generation chephalosporin**				**Carbapenem**	
	**Year**	**mphA**	**mphE**	**ermB**	**msrE**	**qnrS**	**qnrB**	**CTX-M 27**	**CTX-M 15**	**DHA**	**CMY**	**VIM**	**NDM-5**
FC 1026	2025	+				+	-	-	+				
FC 1187	2024	+				+	-	+	-				
FC 1640	2017	+				+	-	+	-				
FC 1795	2021	+		+		+	-	-	-		+		
FC 2232	2024	+		+		+	-	-	-		+		
FC 2239	2018	+				+	-	+	-				
FC203	2024	+				+	-	+	-				
FC399	2024	+				+	-	+	-				
FC603	2023	+				+	-	-	+				
FC838	2023	+				+	-	+	-				
FC1039	2023	+				+	-	+	-				
FC1419	2024	+				+	-	-	+				
FC1428	2018	+				+	-	+	-				
FC1505	2024	+				+	-	+	-				
FC1625	2024	+				+	-	+	-				
FC1663	2024	+				+	-	-	+				
FC1719	2023	+				+	-	+	-				
FC1940	2023	+				+	-	-	+				
FC2365	2024	+				+	-	+	-				
FC2385	2024	+				+	-	+	-				
FC2906	2021	+				-	+	-	-	+			
FC2973	2016	+				-	+	-	-	+			
FC3008	2016	+				-	+	-	-	+			
FC3136	2022	+				+	-	+	-				
FC1903	2020	+				+	-	+	-				
KFC1278	2024	+				+	-	+	-				
FC1906	2021	+				-	+	-	-	+			
FC1860	2023	+				+	-	-	+				
FC1455	2015	+		+		-	-	-	+				
FC1582	2022	-	+		+	_	-	-	-			+	
FC1738	2024	+				+	-	+	-				
FC18	2023	+				-	+	+	-				
FC564	2025	+				+	-	+	-				
FC46	2025	+				+	-	-	-				
FC128	2025	+				-	+	+	-				
FC124	2025	+				+	-	+	-				
FC853	2017	+				+	-	+	-				
FC1446	2017	+				+	-	+	-				
FC1614	2017	+				+	-	+	+				
FC3115	2019	+				+	-	+	-				
FC3225	2021	+				+	-	+	+				
FC2492	2018	+				+	-	+	+				
FC3080	2018	+				+	-	+	-				
FC1368	2025	+				+	-	+	-	+			

FC470	2015	+				-	+	-	-	+			
FC2206	2023	+				-	-	-	-				+
		Isolates obtained from collaborating centers											
FC 101	2025	+				+	-	+	-				
SC7	2025	+				+	-	-	+				
SC7A	2025	+				+	-	-	+				
SC8	2025	+				+	-	-	+				
SC20	2025	+				+	-	+	-				
FC102	2024	+				-	+	+	+				
